# Pro-democracy platform advocacy: Resisting Big Tech-mediated authoritarianism in Southeast Asia

**DOI:** 10.12688/openreseurope.18820.2

**Published:** 2025-05-08

**Authors:** Mai Van Tran, Tuwanont Phattharathanasut, Haymarn Soe Nyunt, Nalinthip Ekapong, Lewis Young

**Affiliations:** 1 Vrije Universiteit Brussel, Brussels, Brussels, Belgium; 2 Humboldt-Universität zu Berlin, Berlin, Germany; 3 Notes of Workers, n/a, UK; 4 Independent Researcher, Bangkok, Thailand; 5 Intellectum Research Consortium, n/a, UK

**Keywords:** platform governance, transnational advocacy, Big Tech oligopoly, platform-mediated authoritarianism, Global South, Southeast Asia

## Abstract

**Background:**

Global platforms, such as Meta, YouTube, X (formerly Twitter), TikTok, and Telegram, have faced widespread criticisms for facilitating authoritarian repression of dissident voices, especially in the Global South. In response, human rights defenders have increasingly launched advocacy efforts toward the foreign platforms to defend free speech. Despite the varying forms and effects of such transnational efforts, there lacks research that systematically examines their dynamics.

**Methods:**

This study advances a concept of **pro-democracy platform advocacy** and scrutinises ***the extent to which such advocacy might affect Big Tech’s practices and curb platform-mediated repression*** in the Global South. The comparative empirical evidence comes from Myanmar, Thailand, and Cambodia, as there exist similar combinations of digital repression while the human rights advocates adopt varying advocacy approaches during 2020–2024. We conduct an exploratory mixed methods analysis of an original dataset of 38 semi-structured expert interviews, 6000 Facebook posts, and relevant Meta’s Transparency Reports.

**Results:**

We find that platform advocacy efforts are more likely to generate significant impact if the advocates focus on issues that resonate with Western democracies, promote campaign publicity via prominent international allies, and are able to engage marginalised dissidents.

**Conclusions:**

The research makes important contributions to both the platform governance and transnational advocacy scholarship by underscoring the unique dynamics of Big Tech governance under authoritarianism in the Global South. Methodologically, by strictly limiting the scope of social media processing to publicly available content with carefully selected accounts and keywords, this study showcases a promising big-data design that minimises privacy risks to vulnerable social media users.

## Introduction: pro-democracy advocacy in an age of Big Tech oligopoly

Existing scholarship on platform-mediated politics has illuminated various platform affordances for either enabling or stifling dissent under authoritarianism ( Lim, 2025; Tufekci, 2017). It has further disaggregated regimes’ and dissidents’ innovative efforts to take advantage of specific affordances for their political ends ( Bradshaw *et al*., 2021; Freedom House, 2020; Roberts, 2018; Ryan & Tran, 2024). Authoritarian leaders have increasingly attempted to shape such affordances themselves, by ordering or influencing major platform companies, such as Meta, YouTube, X (formerly Twitter), TikTok, to censor or suppress the visibility of dissident content ( Global Network Initiative, 2023; Jiang, 2014; Kaye, 2018). These issues in the Global South are exacerbated by the tech giants’ negligence for meaningful reflections as they mainly focus on appeasing U.S. and European public opinion ( Douek, 2021; Khan, 2021; LICADHO, 2021). According to Douek (2021), the reports that Facebook commissioned on human rights impact assessment in developing countries combined “do not match the length or depth of the Civil Rights Audit Facebook commissioned and released about its impact in the United States” (59). The Big Tech companies have also been accused of “barely investing in harm assessment and mitigation outside of the U.S. and English language content” ( The Global Coalition for Tech Justice, 2024).

In response, pro-democracy activists have launched multiple advocacy efforts toward their own governments as well as foreign platforms in order to improve safeguards for free speech. Our study advances a concept of **pro-democracy platform advocacy** that encompasses these advocacy efforts toward social media giants with an aim to defend free speech against digital repression. Most of the advocacy efforts have directly called out the platforms’ *lack of accountability*, urging them to provide more effective remedy and transparency to users. Examples include calls for the platforms to make platform policies, functions, and transparency reports available in local languages; provide account safety features and physical protection to vulnerable users and victims of abuse; provide more user-friendly appeal options; and grant access to data on platform actions to vetted actors and international judicial bodies ( Access Now, 2021; Access Now, 2022; Khan, 2021; Kharroub, 2022; La Rue *et al.*, 2010; The Global Coalition for Tech Justice, 2024; Vialle *et al.*, 2022). Moreover, many advocacy efforts have also focused on Big Tech’s *lack of moderation capacity* toward abusive content, recommending them to invest more resources in smaller markets or politically unstable societies. For instance, the recommendations include: to engage with local civil society representing vulnerable users; staff content moderation teams with native speakers who are familiar with local political dynamics; and keep community guidelines updated with the changing landscape of authoritarian abuse ( Access Now, 2021; Access Now, 2022; Internews, 2023; The Global Coalition for Tech Justice, 2024; Vialle *et al.*, 2022). Last but not least, to tackle platforms’ *promotion of authoritarian propaganda* and *lack of protection for dissident content*, civil society actors have asked platforms to actively amplify high-quality ethical journalism; revise any overbroad definitions of violating content; and mount legal challenge against autocrats’ removal requests ( 7amleh, 2020; 7amleh *et al.*, 2023; Amnesty International, 2020; ARTICLE 19 *et al.*, 2023; Hao, 2021).

In response to such advocacy, the platforms have at times taken action to improve accountability, content moderation, or curation. The majority of the responses have been quick fixes, including removing individual content or accounts that human rights advocates flagged as abusive, using hash databases to automatically detect and prevent re-uploads of previously flagged content, or organising digital literacy training for civil society and human rights defenders operating in authoritarian contexts. However, some have implemented more structural changes, such as, prioritising content from trusted news organisations, allowing users to update their “ad preferences”, hiring more staff familiar with local languages and contexts to actively moderate, engaging in cross-platform collaborations to tackle violent extremism, or establishing a mechanism for independent oversight of human-rights based content moderation ( Meyer *et al.*, 2020; Ryan & Tran, 2024).

Despite the varying forms of pro-democracy platform advocacy and their effect on platform practice, there lacks research that theorises and systematically examines variation in the effectiveness of these efforts. Building upon insights from platform governance and transnational advocacy scholarship and employing a mixed methods research design based on original evidence from Southeast Asia, this study examines ***the extent to which pro-democracy platform advocacy might affect Big Tech’s practices and curb platform-mediated repression*** in the Global South. Our findings suggest that such advocacy is more likely to have a broad and lasting impact on Big Tech-mediated repression under three conditions: first, when advocates align their demands with the platform governance priorities of Global North democracies, i.e. the West; second, when they can collaborate with influential international partners to amplify their messages; and third, when they are able to prioritise the perspectives of marginalised dissidents.

The following section reviews the relevant literature on platform governance and transnational advocacy and demonstrates their shortcomings in explaining the evolving landscape of platform governance under authoritarianism in the Global South. Then, we introduce a novel theoretical framework that highlights the role of Global South advocates’ external engagement and advocacy issues’ resonance with the West for effective platform advocacy. Afterwards, we present a mixed methods empirical design to examine the proposed framework with an overview of our semi-structured expert interviews and social media data. This is followed by data analysis and discussion of findings.

Overall, the research elevates the global relevance of the platform governance scholarship by highlighting the oft-neglected experiences and political agency of millions of internet users living under authoritarian regimes. It also deepens our understanding on transnational advocacy by demonstrating the unique dynamics of pro-democracy advocacy in an age of Big Tech oligopoly in the Global South.

## Literature review

Social media giants, such as Meta, YouTube, X (formerly Twitter), TikTok, and Telegram, play an influential role in mediating political communication across democracies and autocracies alike. According to the latest World Values Survey, about 45% of its respondents access domestic and international news via sites like Facebook and Twitter on a daily basis ( Haerpfer *et al.,* 2022). Zooming in on autocracies, these global platforms have faced widespread criticisms for facilitating authoritarian repression of dissident voices, especially in the Global South. Current scholarship on platform governance has highlighted both the platforms’ vulnerability to authoritarian abuse as well as governments’ effort to directly regulate online content ( Bradshaw *et al.*, 2021; Sombatpoonsiri & Mahapatra, 2024). Yet, little analysis exists on interactions between dissidents and platform companies, particularly regarding advocacy efforts by pro-democracy civil society to demand Big Tech to counter digital repression across their sites.

The first well-publicised case of platform advocacy in the Global South was Myanmar civil society’s 2018 open letter toward Mark Zuckerberg accusing Facebook of enabling anti-Rohingya violence due to its lack of effective monitoring over hate speech and violence incitement content. The civil society actors then urged Facebook to invest more resources into content moderation, engage local groups, and improve transparency of its processes and interventions ( Phandeeyar *et al.*, 2018). Other communities similarly experiencing active conflict or growing authoritarianism, from Palestinian to Hong Kong netizens, have also recently called on platforms to address its censorship of dissident voices as well as inaction toward hate speech and violence incitement ( 7amleh *et al.*, 2023; ARTICLE 19 *et al.*, 2023). As *platform advocacy* has a growing presence in pro-democracy activists’ repertoire in the digital age, it is imperative that we address our knowledge gap on this phenomenon.

### Platform governance in authoritarian, Global South contexts: The missing agency of pro-democracy civil society

Existing analysis on civil society’s advocacy toward Big Tech has mainly come from platform governance studies. Gorwa (2019) conceptualises platform governance as comprising of “legal, political, and economic relationships structuring interactions between users, technology companies, governments, and other key stakeholders in the platform ecosystem” (2). However, most of the growing scholarship that has examined the transnational and multistakeholder nature of “state – civil society – platform” interactions focuses on Global North democracies, with little to say regarding the Global South, let alone Southern autocracies ( Gorwa, 2019; Papaevangelou, 2021). Latest studies on the typology and role of civil society actors at global Internet multistakeholder institutions lack dedicated attention to the experiences of Southern actors ( Mueller, 2010; Radu *et al.*, 2023; Tjahja *et al.*, 2021). There is only some initial recognition for the barriers to Southern civil society’s participation at these multistakeholder venues, including technical knowledge, financial funding, cultural and language differences ( DeNardis, 2014). Studying representation of civil society at the annual RightsCon conference, a leading multistakeholder venue with participation by policy makers and practitioners on human rights in the digital age, Grover (2022) finds that “interests from the Global North and West are highly over-represented” with a lot more sessions on privacy and misinformation than on internet shutdown (1) – a pressing issue during mass contention or violent conflict in the Global South.

Therefore, a lacuna exists regarding understanding on the political agency of *pro-democracy civil society* as well as their advocacy influence on Big Tech’s policy and practice in Southern autocracies. First, since the tech giants are headquartered mainly in the Global North, it is important to understand how Southern civil society actors might engage with these companies despite the lack of accessible and conducive channels. While Global North governments and their civil society might struggle to regulate Big Tech, their wealthy economies turn them into the most important stakeholders for these multinational corporations. As Lim (2025) argues, social media operate under the logic of platform capitalism that prioritises “the acquisition and monetization of user data” (7). Hence, Global North societies, by controlling Big Tech’s ability to access the most lucrative markets, possess much more capacity to influence the companies’ policies than the Global South ^1^.

Second, within the Global South, there is wide variation in terms of socio-economic development and political regimes. While existing platform governance scholarship’s findings on Northern state-civil society dynamics might generalise to liberal regimes in the Global South, they have limited applicability for more illiberal contexts ( Ong *et al.*, 2024, 40–44). Among illiberal regimes, Big Tech platforms appear more likely to accommodate law enforcement’s takedown requests or create moderation exceptions for government-sponsored content if such governments control sizeable markets ( Sombatpoonsiri & Mahapatra, 2024). This might create an intentionally hostile environment against pro-democracy platform advocacy. Yet, human rights advocates in poorer autocracies whose markets generate less revenue for Big Tech also face a major yet different challenge in being less able to gain attention by decision makers at these profit-oriented companies, let alone influence them. Indeed, an important aspect that existing studies have yet to examine is how these advocacy efforts have evolved despite autocrats’ active agenda to weaken and silence any types of activism that they perceive to threaten their rule. 

So how might Global South advocates overcome these compounded challenges and manage to shape Big Tech practice to curb digital repression? The more established literature on transnational advocacy, which we turn to next, offers more insight on the operating environment and agency of marginalised civil society actors under Southern autocracies.

### Transnational advocacy from the Global South: Big Tech as a qualitatively different target

Keck and Sikkink (1998) characterise transnational advocacy as consisting of specific advocacy campaigns launched by transnational networks of actors, “who are bound together by shared values, a common discourse and dense exchanges of information and services” (2). Most literature on the Global South has conceptualised such *networks* as North-South and their *targets* as Global South states ( Keck & Sikkink, 1998; Piper & Uhlin, 2004; Young, 2023). As the scholarship originates from contexts where civil society in Southern autocracies face repression, it highlights how these actors call for support from civil society allies in Northern democracies, who then lobby their own democratic governments to put pressure (e.g. economic sanctions or international condemnation) on the Southern autocrats into implementing political liberalisation. Southern civil society would initiate such *boomerang* strategies when they expect benefits from transnational networks, such as “access, leverage, and information (and often money) they could not expect to have on their own” ( Keck & Sikkink, 1998, 12–13).

However, this state-centric model might not adequately explain the dynamics of pro-democracy platform advocacy. The growing capacity of Big Tech in influencing politics across borders has turned these multinational companies into important advocacy targets themselves. Indeed, *pro-democracy advocacy* toward Big Tech has qualitatively different networks and targets compared to what the conventional transnational advocacy framework theorises.

First, the transnational nature of pro-democracy platform advocacy is mainly due to the Global North bases of advocacy targets, i.e. social media giants. While Global South civil society might still solicit support by Global North actors to induce international leverage, Global North governments might play a less important role. This is partly because a Northern state is unlikely to exert leverage on social media giants for their harmful actions outside of the state’s territory or without direct impact on its citizens. Besides a potential lack of interest, there exists limited legal basis that warrants a state’s capacity to punish their multinational enterprises for facilitating human rights abuses abroad ( Wolfsteller & Li, 2022). Moreover, global human rights advocates have largely urged states to carefully limit circumstances where they might hold social media platforms legally responsible for harmful or illegal user-generated content, since this might risk infringing upon freedom of expression ( APC, 2023; Electronic Frontier Foundation *et al.*, 2015). Hence, the pre-dominant model of transnational advocacy that focuses on Southern autocrats as advocacy targets and emphasises the role of Western states’ leverage might not travel well to the contexts of pro-democracy platform advocacy.

Second, different from other types of multinational corporations from the Global North that also operate in Global South authoritarian states (such as big oil or apparel conglomerates), tech giants' products can directly penetrate the daily lives of ordinary local residents via the Internet without these companies having to set up dedicated operations inside most of the countries. Hence, their operations are much more out of touch, offering few or no easily accessible physical contacts for the residents. As a result, Global South social media users are less able to directly confront the tech giants with their grievances, in contrast to a wide range of grassroots advocacy toward other Global North businesses on labour exploitation or environmental destruction ( Caraway, 2006, 292–293; De Winter, 2001, 102; Pye, 2023, 335; Rodrigues, 2011, 5). Platform advocacy, therefore, is less likely to benefit from active engagement by the victims of company-enabled harm and more likely to be directed by civil society elites, who are human rights advocates with experience in transnational advocacy acting as the victims’ *allies*. These advocacy actors are aware of the respective censorship cases on social media platforms and motivated to tackle the abuse. They act as core campaigners who endeavour to represent censorship targets while simultaneously launching engagement with social media platforms.

Nonetheless, despite these crucial distinctions, there are some nuanced insights from the existing literature that could inform our understanding on the civil society actors that launch pro-democracy platform advocacy campaigns. As Piper and Uhlin (2004) astutely observe in their edited volume on transnational activism in Asia, the conventional boomerang model might have more validity in some authoritarian contexts than others. This is because in cases where regimes are highly repressive, it is challenging for any pro-democracy activism to emerge, let alone connect with sympathetic allies abroad to launch transnational advocacy. In such cases, advocacy campaigns are usually initiated by foreign civil society without meaningful embeddedness, i.e. integration into and leadership from the “directly impacted community” ( Ciplet, 2019, 300). Even when local activists participate in transnational advocacy, in many cases, they “tend to be well-educated, middle-class people” who might have limited capacity to represent grassroots interests of marginalised communities ( Piper & Uhlin, 2004, 17). In addition, due to the lack of domestic funding for pro-democracy activism, only civil society actors who manage to obtain financial backing from Western foreign aid are able to take part in transnational advocacy ( Young, 2023). This might have implications for platform governance in the Global South where the advocacy campaigns are susceptible to being launched externally and lacking meaningful participation by the affected end-users. In the next section, our study proposes an original theoretical model to account for variation in the impact of platform advocacy from the Global South.

## Theorising pro-democracy platform advocacy

We propose that the impact of pro-democracy platform advocacy depends on its (1) advocacy issue’s resonance with the West and (2) advocacy actors’ engagement with (a) prominent international allies and (b) marginalised local dissidents.

First, how might the advocacy agenda by Global South advocacy actors gain attention and induce response by the tech giants? We hypothesise that this depends on the support by internationally prominent allies to amplify the Global South advocacy demands and Western donor funding to sustain the advocacy efforts. In particular, international allies such as global mass media companies or established human rights INGOs can play an important role in pushing for platforms’ policy change by affecting the companies’ reputation worldwide. Hence, the more active the Global South advocates are in engaging with the international allies, the more impact their advocacy might generate. Moreover, advocacy impact also depends on the campaigns’ durability, i.e. the ability of Global South advocacy actors to continuously document issues and follow up with Big Tech. In order for such advocacy to sustain and grow, Western donor funding (mainly from the U.S., EU, U.K., and Australia) plays a major role in financing their operations ( Young, 2023).

Nonetheless, platform advocacy might receive low level of global attention if they come from states with low level of economic development and low “policy relevance” to the West ( Ramos *et al.*, 2007; Ron *et al.*, 2005). Moreover, the advocacy actors might find it challenging to raise funds as resisting platform-enabled repression is of a lower priority in Western donors’ international development agendas. So which issues might motivate the international allies and Western donors to support pro-democracy platform advocacy? We hypothesise that platform advocacy from the Global South is more likely to obtain foreign funding and attract interests from prominent international organisations if the advocacy issue resonates with main platform governance priorities in Western democracies. Indeed, social media platforms pose certain similar issues across societies, including online violence and inauthentic information operations. Hence, advocacy efforts that centre their demands around combating electoral disinformation (e.g., #YearOfDemocracy campaign) or online harassment (e.g., #FightOnlineAbuseNow campaign) might gain more international support than those that call for platforms to challenge governments’ content takedown requests, tolerate violent conflict-related content, or demote autocrats’ electoral campaigns.

Second, how might the advocacy efforts address the widest range of platform-mediated repression cases? We hypothesise that this mainly depends on the ability of the advocates to actively cultivate connections with marginalised dissidents. Due to existing discriminatory and misogynistic norms, female, LGBTQ+, rural, or ethno-religious minority dissidents have become more vulnerable to autocrats’ identity-based online abuse campaigns and Big Tech’s lack of accountability ( LICADHO, 2021; Myanmar Witness, 2023; Sombatpoonsiri *et al.*, 2023). Hence, targets of censorship who suffer multiple layers of marginalisation, such as rural ethnic minority women in highly repressive regimes, might be less represented in platform advocacy and face more challenges in holding platforms accountable. As a result, the more inclusively embedded the advocates’ networks are, the more representative their advocacy might become and the larger its potential impact.

**Figure 1.**
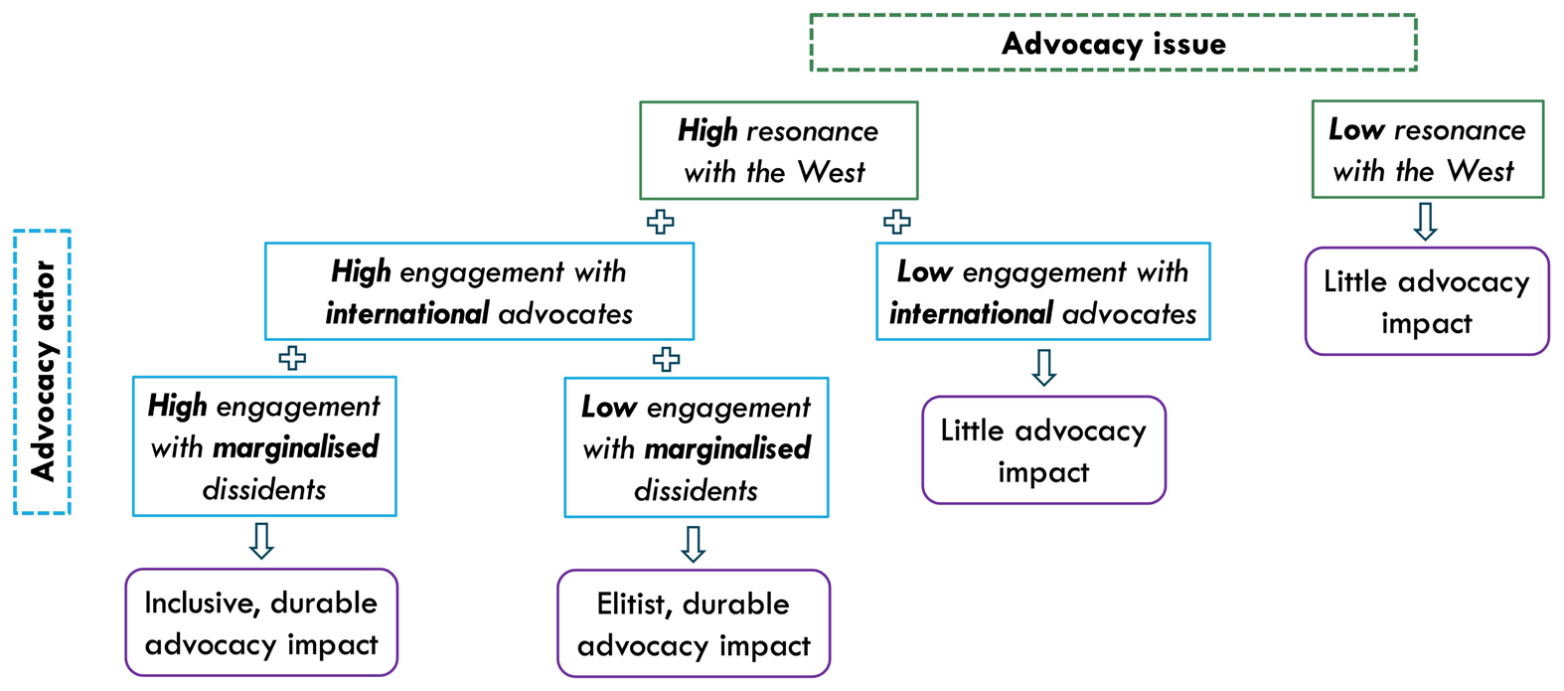
Proposed framework of pro-democracy platform advocacy.

Overall, we propose that pro-democracy platform advocacy efforts are more likely to foster Big Tech’s accountability to dissident voices and curb platform-enabled repression if their demands resonate with Western concerns and if they actively promote international publicity (via prominent allies) and cultivate rapport with local marginalised communities ( Figure 1).

## Methods

### Comparisons across Southeast Asia

**Figure 2.**
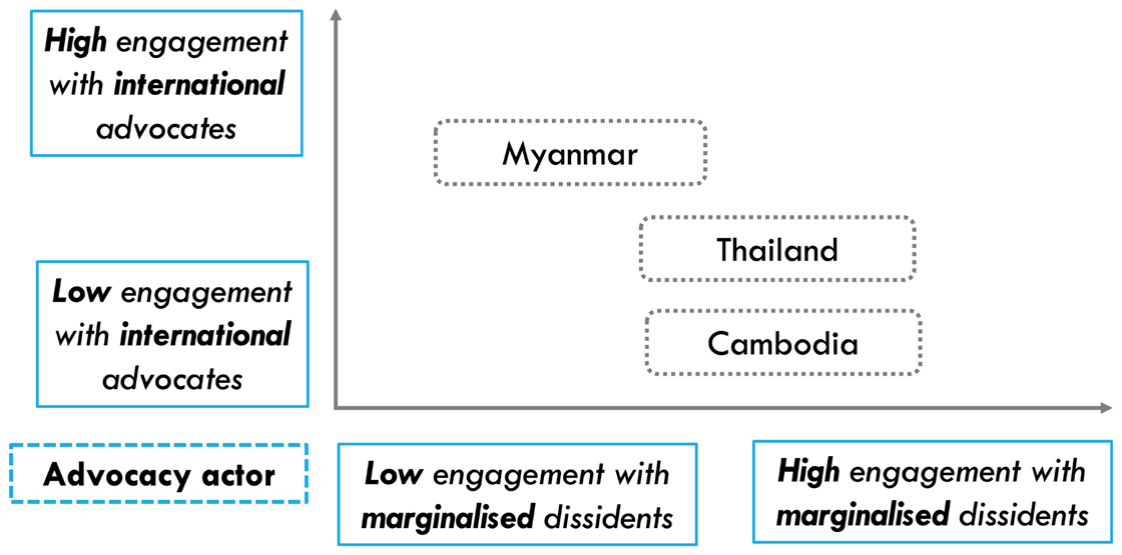
Variation in platform advocates’ external engagement in Myanmar, Thailand, and Cambodia.

To examine our theoretical framework, we present comparative evidence from Myanmar, Thailand, and Cambodia, where autocrats constantly attempt to control and abuse social media, including Big Tech platforms. On the one hand, to analyse the role of advocacy issue’s Western resonance for effective advocacy, we will conduct within-case comparisons based on original evidence of different platform advocacy efforts from Myanmar and Thailand. On the other hand, to identify the impact of advocacy actors’ external engagement on advocacy outcomes, we will conduct cross-case comparisons as the three countries experience similar types of platform-enabled repression while their advocacy actors have varying approaches on external engagement ( Figure 2).

### Three comparative cases

In the past decade, along with the growing dominance of social media giants in mediating political communication in Asia, illiberal politics has also expanded across this continent – from shrinking civic space in democracies to executive aggrandisement in autocracies and more drastic regime change via military coups ( Kasuya & Wang, 2024; Staniland & Vaishnav, 2023). Southeast Asia itself has witnessed a notable decline in internet freedom, with a regional average score dropping from 48.6 in 2015 to 41.9 in 2024, according to Freedom House’s data (where a score between 40 and 69 is considered “partly free”). Authoritarian repression increasingly relies on or benefits from popular Big Tech platforms’ infrastructure. This is particularly the case Myanmar, Thailand, and Cambodia. In response, their pro-democracy activists have launched multiple transnational advocacy efforts toward the tech giants to demand human rights-based conduct.

***Myanmar.*** Myanmar has experienced one of the most violent civil conflicts in the world since its 2021 military coup while major digital platforms (such as Meta, TikTok, YouTube, Telegram, and Viber) play an active role in mediating its online public sphere. Not only has Facebook monopolised Myanmar’s social media landscape and but it has also become the definition of the Internet across the country ( Whitten-Woodring *et al.*, 2020). Unsurprisingly, it is considered an effective tool for the Myanmar political leadership to mobilise mass violence, including spreading anti-minority hate speech and promoting large-scale communal violence, especially in the lead up to the Rohingya Crisis in 2017. Hence, parallel to the troubling authoritarian abuse of Facebook, digital rights advocates have continuously launched advocacy campaigns to expose the Myanmar authorities’ influence operations as well as condemn Meta for facilitating violence incitement ( Ryan & Tran, 2024).

After the 2021 military coup, not only has the State Administrative Council (SAC), i.e. the coup leaders, but also anti-SAC armed forces have relied on Facebook, YouTube, TikTok, Telegram, and Viber to mobilise violence as well as spread propaganda to gain popular support and discredit their enemies ( Myanmar Internet Project, 2024; Ryan *et al.*, 2024). While the SAC has throttled the use of Meta platforms inside the country by both banning the platforms and the use of VPNs, other apps such as YouTube, TikTok, Telegram, and Viber remain accessible. Digital rights advocates repeatedly demanded these platforms to invest more resources in content moderation ( Myanmar Internet Project, 2024; Myanmar Witness, 2023). Global Witness (2021) called out Facebook’s active recommendation of pro-SAC accounts. Myanmar ICT for Development Organization together with international media urged TikTok to moderate SAC soldiers’ threatening videos ( Guest *et al.*, 2021; Reuters, 2021). Local independent media and human rights advocates further collaborated with international media and UN representatives to urge Telegram to moderate anti-dissident disinformation, doxing content, and violence promotion on its platform ( Anlezark, 2023; Myanmar Witness, 2023; The Office of the High Commissioner for Human Rights, 2023).

As a result of these advocacy efforts, most of the targeted platforms have stepped up their moderation of violent content from Myanmar’s armed actors, with Meta being the most active in banning SAC-linked accounts and moderating abusive content while Telegram and Viber being the least invested ( Global Witness, 2021; Guest *et al.*, 2021; Myanmar Internet Project, 2024; Myanmar Witness, 2023). Hence, these authoritarian actors have increasingly migrated to the two alternative platforms to reach their target audience at home and abroad ( DEMAC, 2022, 38; Myanmar Internet Project, 2024).

***Thailand.*** While the Thai authorities tolerate a wider range of political speech than their Myanmar or Cambodian counterparts, Thailand’s former military government (2014–2023) frequently requested Big Tech platforms to take down content accused of being anti-monarchy. Moreover, the regime’s grassroots supporters actively coordinated pro-military and pro-monarchy propaganda as well as anti-dissident smear campaigns ( Asia Centre, 2022; Asia Centre, 2023a; Freedom House, 2023b; Sombatpoonsiri *et al.*, 2023). In 2023, Thailand is ranked 3rd in Google’s global removal requests regarding government criticism content, with almost 100% compliance by the company affecting more than 800 items (data extracted from Google’s Transparency Report for 2023) ( Google, n.d.). In the second half of 2023 alone, Meta also complied with the government’s requests to restrict access to more than 800 items for “allegedly violating lese-majeste law” ( Meta, n.d.). Similarly, TikTok complied with almost all of the government’s content takedown requests regarding around 500 items in 2023 ( TikTok, 2023; TikTok, 2024). Moreover, Big Tech lack measures to tackle known risks of disinformation. For instance, Meta did not have any guidelines for political advertisements for the 2023 Thai election ( Arugay & Sriyai, 2023).

In response, digital rights advocates in Thailand have called on the platforms to publicise detailed reports on the government’s takedown requests ( Asia Centre, 2022; Asia Centre, 2023b); grant access to data on digital repression to local civil society actors ( Sombatpoonsiri *et al.*, 2023); and engage with local partners to raise public awareness on media and information literacy ( Asia Centre, 2023a). They have further urged Big Tech to update content moderation strategies toward both anti-dissident content and accounts ( Asia Centre, 2023b; Sombatpoonsiri *et al.*, 2023). Nonetheless, the platforms’ responses have been inconsistent. For example, in 2020, due to international criticism against their compliance with a government request to take down the anti-monarchy Royalist Marketplace group ( Amnesty International, 2020), Meta did not comply with the following request to take down the second version of this group. In 2020 – 2021, Meta and Twitter further took down a network of pro-regime accounts involved in coordinated information campaigns that human rights advocates reported, but not necessarily the clone accounts ( Asia Centre, 2023a; Goldstein *et al.*, 2020). Then, in the lead up to Thailand’s 2023 elections, Meta, Google, and TikTok worked with CoFact, a local fact-checking network, to provide digital literacy training ( Asia Centre, 2023c).

***Cambodia.*** While Big Tech, especially Meta, have been credited with facilitating pro-democracy and human rights advocacy in Cambodia ( Young, 2023), these platforms have also enabled digital repression. Pro-regime supporters and media have actively engaged in coordinated harassment and disinformation campaigns, particularly via Facebook, Twitter, TikTok and Telegram, against regime dissidents ( Cambodian Center for Human Rights, 2023; Norén-Nilsson, 2021; Sopheanut, 2024). This has resulted in a high level of self-censorship among regime dissidents as well as a highly pro-regime digital information environment ( Cambodian Journalist Alliance Association, 2023).

The first and only major platform advocacy effort so far was by the Cambodian League for the Promotion and Defense of Human Rights in 2021 toward Meta to demand moderation of anti-dissident content and improved safety features ( LICADHO, 2021). There was no direct response by the company. However, in the lead up to Cambodia’s 2023 election, Meta removed a pro-regime influencer’s live video that contained death threats against an independent journalist in exile in the U.S. ( Cambodian Center for Human Rights, 2023). Yet, during the same period, when Meta’s Oversight Board recommended a temporary suspension of former Prime Minister Hun Sen’s account due to violence incitement content, Meta’s executive rejected the recommendation ( Access Now, 2023; Freedom House, 2023a).

### Data collection and analysis methods

To scrutinise the varied dynamics of pro-democracy platform advocacy across the three cases, our research adopts an exploratory mixed methods design. First, during December 2023 – March 2024, with purposive sampling, we conducted semi-structured expert interviews with 38 respondents from Myanmar, Thailand, and Cambodia, who either engaged in platform advocacy (toward Meta, Twitter, YouTube, TikTok, or Telegram) during 2020–2024 or represented marginalised dissidents (female, LGBTQ+, ethno-religious minority, or rural activists). The interview questions focused on their experiences with conducting online activism, encountering platform-enabled repression, or participating in any platform advocacy efforts ( Tran *et al.*, 2024). Then, based on insight from the interviews, we built a list of prominent political Facebook accounts and keywords to capture anti-dissident content from public Facebook pages and groups across the three societies in March - April 2024 ^2^. As discussed above, not only does Facebook continue to be the most popular platform in the three cases, it has also been the main site of authoritarian abuse and target of civil society’s advocacy.

Based on our interviews, desk research, and team members’ expertise, we first looked for public Facebook accounts or keywords associated with Myanmar, Cambodian, and Thai authoritarian content, including (a) pro-regime propaganda, (b) anti-dissident disinformation, (c) promotion of anti-dissident violence, or (d) anti-dissident doxing content. While (a) pro-regime propaganda would portray the autocrats with a positive tone or use a negative tone toward dissidents, (b) anti-dissident disinformation would promote false information about dissidents in order to discredit them. Other authoritarian posts might also (c) promote or coordinate violence toward dissidents or (d) publicise their private information, photos or videos to harass or enable arrests against them. Following most of the platforms’ community guidelines, we classify disinformation, violence promotion, and doxing as abusive content. Our team worked together over a month to test the Facebook accounts and keywords to make sure we cast the net widely enough to capture relevant content while not being too generic so as to avoid capturing mainly irrelevant content.

Over the two months of March and April 2024, we used CrowdTangle – Meta’s own social media monitoring tool – to capture content on a daily basis based on our list of authoritarian accounts and keywords. This has resulted in an original dataset of more than 230,000 public Facebook posts. We then manually coded and analysed the prevalence of authoritarian content in three random samples of: 2000 Myanmar posts, 2000 Cambodian posts, and 2000 Thai posts ( Tran *et al.*, 2024). First, we coded for the presence of the four subtypes of authoritarian content in each post. Second, we coded for the presence of marginalised targets, i.e. female, LGBTQ+, ethno-religious minority, or rural dissidents. Among our team members, two researchers with expertise in platform-mediated politics in each country case coded content together from the corresponding case. The lead author also regularly reviewed the coding and provided feedback during our iterative coding process in order to ensure high inter-coder reliability and consistent operationalisation of the coding categories across the three cases. Finally, we juxtaposed our primary analysis on Facebook’s moderation practice with Meta’s own Transparency Reports during 2020–2024.

While the interview analysis would provide qualitative evidence on the role of issue resonance and external engagement for impactful platform advocacy, the quantitative social media data analysis would triangulate these findings. First, regarding issue resonance, while government-sponsored anti-dissident information operations (IO) and content takedown requests are both prominent issues of platform-facilitated repression in Thailand, due to the former’s higher resonance with the West’s focus on online disinformation, we expect there would be more donor funding for civil society’s anti-IO work. If so, our analysis of Meta’ transparency reports should demonstrate that, as a result, there is more consistent effort by Meta to address IOs than to resist takedown requests by the Thai authorities. Second, since Myanmar human rights advocates have engaged in more international advocacy campaigns around anti-dissident information operations compared to their Thai and Cambodian counterparts ( Figure 2), we expect that authoritarian content is less prevalent and more frequently removed on Myanmar Facebook compared to Thai and Cambodian Facebook. Third, as most Myanmar advocacy actors have fled their country after the 2021 coup, they might face more physical challenges than their Thai or Cambodian counterparts in reaching out to marginalised dissidents outside of their existing networks. Hence, we expect that authoritarian content targeting marginalised dissidents are more prevalent and less moderated in Myanmar. Together, the findings would demonstrate achievements and challenges in Global South advocacy efforts against platform-mediated authoritarianism.

## Findings

### Issue resonance with the West: Hate speech and disinformation vs. The rest

Impactful platform advocacy requires long-term monitoring of the evolving patterns of platform-mediated repression and sustained pressure on Big Tech to address harmful practice. A political analyst stressed the importance of maintaining persistent advocacy on the same issues:

In terms of the policy recommendations that we publish in our reports, it’s not a matter of one report’s recommendations. It’s a matter of many reports, many recommendations, plenty of these messages that we try to convey through these recommendations sinking into their minds. And they can start saying there is something that needs to be changed. (Interview 32)

Yet, many respondents acknowledged how their current monitoring suffers from a low level of technical capacity in data processing (Interview 1, 3, 28, 32, 34) and a lack of funding to continue their existing efforts in the long run (Interview 34). A Thai digital rights advocate emphasised: “Digital harassment evolves and becomes more sophisticated over time. You need to sustain this effort on data collection. If you do it for 3–5 years, it doesn’t have substantive impact” (Interview 34).

In particular, not all advocacy issues receive the same level of international prominence, and hence funding. This is particularly the case for many pro-democracy demands for Big Tech’s accountable practice in Global South conflict areas that might be at odds with practice considered to defend democracy in the West. Indeed, Myanmar’s human rights advocates might push for seemingly the opposite of what their Western counterparts promote. After the 2021 coup, Myanmar advocates asked Meta not to raise awareness on an upcoming election, since it is organised by the SAC and likely to be fraudulent:

Last year, we had a meeting with [Meta staff] talking about the policy around the junta-planned election. We explained why it’s important that the election shouldn’t be legitimised by Facebook. I knew they were prepared for the election, including putting people who’re running in the electoral candidate category. [...] But we explained this is a different matter in Myanmar. (Interview 20)

In another case, many Myanmar human rights advocacy groups have been unable to promote Facebook content to their target audience after they went into exile. This is because the platform strictly bans foreign interference: “If I’m in Thailand, I can’t use the Facebook boost for our social media campaigns for Myanmar audience. [...] And those in Myanmar don’t want to be the main contact for our social media campaign [due to security concern]” (Interview 20). They were not able to persuade Meta to create an exception. Similarly, a rural independent media organisation in Myanmar could not convince Meta to be more flexible with its policy around moderation of graphic violence content – a policy that might have censored important war-time updates:

We had a discussion with Facebook’s Myanmar representatives about how Facebook should not ban all the posts with bloody pictures or shooting automatically or demote page quality from green to yellow. Facebook said that if we publish those pictures again, our page can be shut down. Sometimes it’s hard to find words to describe the cruelty of the military, but people can see visually, so we want to use pictures as evidence. But we can’t post on Facebook. Facebook said they need to take care of other international communities and these photos aren’t suitable for them. [...] They don’t focus on Myanmar users, instead they standardise and make restrictions globally. (Interview 16)

None of these demands for Big Tech to create exception in their content policy received further amplification by Myanmar advocates’ international allies, let alone funding for advocacy campaigns. This stands in stark contrast to highly visible international backing for Myanmar civil society’s monitoring and advocacy around online hate speech and disinformation. Indeed, due to sustained international advocacy campaigns on Facebook’s enabling of hate speech in Myanmar since the 2017 Rohingya Crisis, Meta has significantly increased engagement with Myanmar human rights organisations to provide case-based support as well as implemented structural changes to content moderation. First, ad-hoc support to local civil society includes engaging human rights advocates as Trusted Partners, helping them and their networks to recover access to hacked accounts (Interview 12, 19), deactivate arrested dissidents’ accounts (Interview 11, 12, 17), or obtain a Verified badge (Interview 15). According to a digital rights advocate’s reflection:

In the past, when people reported to Facebook, it took more than 24 hours to take down or do moderation of content. In a country in a situation like ours, if a content is up for that period of time, particularly if it’s about calling for violence against vulnerable groups, that’s very harmful. So enforcement’s very important. Facebook right now their turnaround rate is much better than before but that doesn’t necessarily mean all reporting’s at that rate. Our escalation for Facebook goes through a separate channel of Trusted Partners. So our escalation will be moderated much faster than others. (Interview 3)

On a more structural level, during 2020–2024, Meta has continued to actively remove politically abusive accounts (Interview 2, 3, 18), conduct more human rights impact assessment, provide periodic briefings to Myanmar civil society, and offer human rights organisations with access to its monitoring tool, CrowdTangle (Interview 3, 11).

The higher level of Western support for more resonant advocacy issues is also visible in the Thai context. While platform-enabled repression in Thailand consistently relies on both (a) takedown requests of dissident content and (b) coordinated anti-dissident IOs, there existed only one case of international amplification of local advocates’ demand for Facebook to resist takedown request ( Amnesty International, 2020). On the other hand, there exist multiple donor-funded reports and campaigns to ask Facebook and Twitter to improve their moderation of pro-regime IOs ( Asia Centre, 2023a; Asia Centre, 2023b; Sombatpoonsiri *et al.*, 2023). One might argue that this disparity is due to Thai human rights advocates’ lower willingness to conduct advocacy against takedown requests since they perceive a higher risk of government retaliation. However, that is unlikely to be the case since pro-regime forces have condemned both types of advocacy, and anti-IO activists have suffered significant amount of digital harassment themselves ( Amnesty International, 2024).

**Figure 3.**
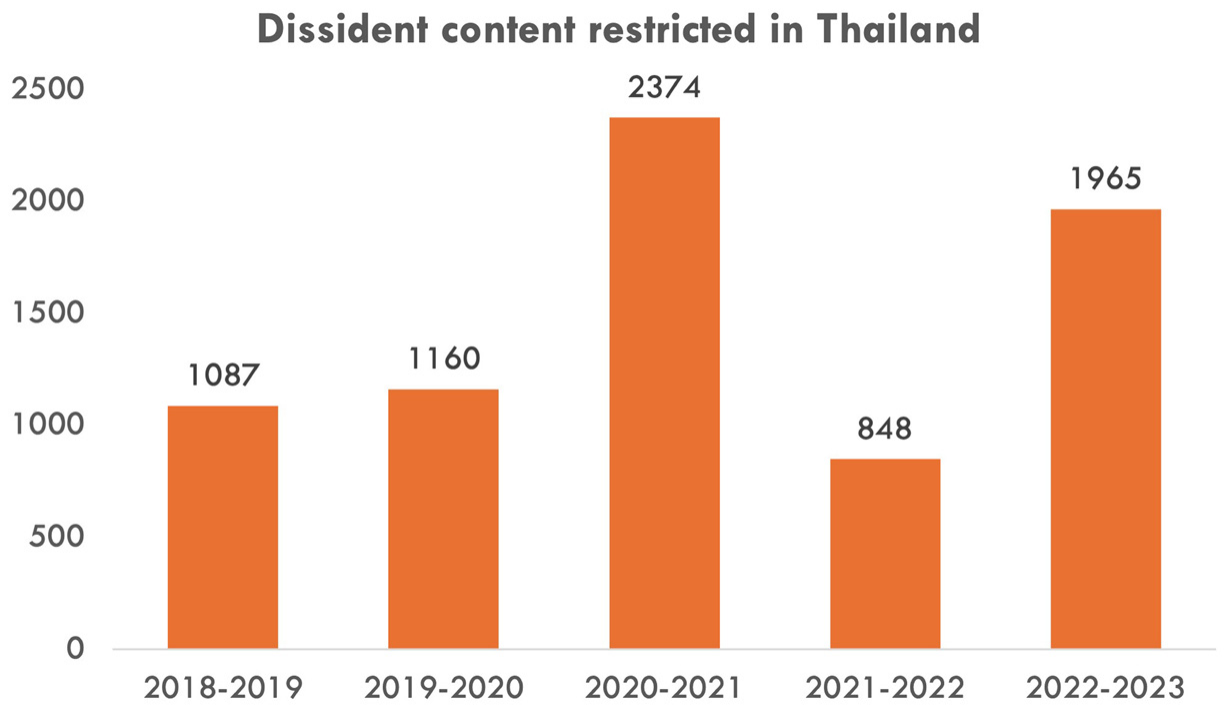
Number of dissident content items Meta restricted in Thailand during 2018–2023.

This difference in the prominence of the two advocacy issues is correlated with Meta’s contrasting levels of effort in addressing them. In particular, while the company has issued multiple reports on content moderation against Thai IOs ( Gleicher, 2019; Harbath & Tan, 2019; Meta, 2021; Osman & Burke, 2021), there exist no reports on any action to challenge the Thai authorities’ takedown request, despite the company’s announcement of this intention in 2020 ( AFP, 2020). Furthermore, Meta’s own reports on content restriction based on Thailand’s local law during 2014–2023 demonstrate how the company continues to restrict domestic access to content alleged by the government to violate the lese-majeste law ( Figure 3) ( Meta, n.d.).

Are Big Tech companies more willing to disrupt authoritarian IOs than to challenge autocrats’ direct orders due to different risk perceptions? This is unlikely to be the case since censoring autocrats’ information campaigns is also a form of public contention and poses equal risks to the companies’ operations in these regimes. After Twitter and Meta accused the Thai military of being involved in anti-dissident IOs and removed these networks, they had to close down their office inside Thailand. Hence, the advocacy issue’s Western resonance appears a more plausible cause for the companies’ variation in responsive actions.

### Amplification by international advocates

Besides Western donor funding, advocacy amplification by international allies plays an important role in enabling Global South demands to solicit Big Tech’s response. Local human rights advocates prefer to stay anonymous due to both *fear* of their autocrats’ reprisal and *expectation* for the prominent international allies’ influence in inducing reactions by Big Tech.

First, the local advocacy actors’ fear of repression is omnipresent across our interviews, including for advocates in the diaspora who are afraid the regimes might revoke their passports or harass their family members back home. One advocate verbalised this concern: “Do [grassroots civil society members] want to take the risk of documenting the violations and calling out the governments? The answer is: most of the time, they don’t want to do that” (Interview 32). Moreover, those that took the risk to publicly condemn digital repression lamented the rarity of Big Tech’s responding to their advocacy. One Cambodian digital rights advocate highlighted the challenges in pressuring companies like Meta to respond to demands that local organisations raised directly via private communications: “When we tried to raise the issue to the platform, it’s really hard to hear back from them. You need to ask many times, sometimes you don’t reach the right people in the organisation, so that made it difficult to be able to work with Facebook” (Interview 28). Similarly, an environmental activist in Cambodia recalls:

I reported [an abusive video to Facebook], no answer at all. The video continued to be there. [...] Then I spoke with a journalist on the topic. [...] So when the journalist asked Facebook about it, Facebook replied to them: “Oh yes, we brought it down, it’s against our policy.” (Interview 24)

Hence, most local advocates expect that amplification by international allies is more likely to induce Big Tech’s response due to the tech giants’ perception of potential reputational damage and subsequent revenue loss on a global scale. According to a Thai digital rights expert:

We want [the Western embassies] to do more when it comes to engaging with platforms. [...] When it comes to content moderation or business and human rights, if you want to make advocacy for these issues work, I think you need allies who platforms care. And as I said, platforms care about markets. The embassies represent states that are then their [main] markets. (Interview 34)

Nonetheless, instead of Western embassies and governments, other international allies such as global media organisations, international human rights organisations, and UN’s independent human rights experts have been much more active in launching their own international advocacy campaigns or holding private meetings with the tech companies to relay the local advocates’ demands (Interview 6, 12, 27, 29, 32, 34). As one digital rights advocate shares:

The INGOs [international non-governmental organisations] definitely have more leverage to use regarding international human rights mechanisms to make policy recommendation, make submissions to the UN’s Universal Periodic Review for example, or publish reports with policy recommendations. And then, some of them might have the network to engage directly with technology companies and make these suggestions. (Interview 32)

Public advocacy for tech accountability in Myanmar has pressured platforms such as Meta, YouTube, TikTok and Telegram to take action. This includes YouTube’s collaboration with Meta to detect malicious actors and participation in civil society’s periodic briefings (Interview 3), TikTok’s removal of SAC soldiers’ violent threats and beginning to engage Myanmar digital rights advocates (Interview 3, 20), and Telegram’s removal of pro-SAC accounts that mobilise violence against anti-SAC dissidents (Interview 1). The case of Telegram further demonstrates the importance of public advocacy via internationally prominent allies. In particular, repeated advocacy campaigns by Myanmar civil society to demand Telegram’s moderation of doxing content during 2021–2022 (Interview 8, 20) led to very limited response by Telegram. According to a signatory of the “Dear Pavel” campaign: “We sent emails, open letters, and policy briefs outlining cases, incidents, and actors that are calling for violence and instigating real-world harm. But so far, we didn’t have any official response from Telegram” (Interview 3). Only when BBC News and the UN’s independent experts amplified the case in 2023 ( Anlezark, 2023; The Office of the High Commissioner for Human Rights, 2023) did Telegram start to have a serious response, removing around 100 violating posts and a dozen of pro-SAC accounts.

In the cases of Thailand and Cambodia, the lower levels of international attention on platform-mediated abuse compared to Myanmar have correlated with less global amplification of local advocates’ demands. As a result, Big Tech’s response to advocacy in the Cambodian case is limited to Meta’s engagement with Cambodian civil society (a) via its Trusted Partner mechanism where very few organisations can directly escalate violations to the platform’s staff (Interview 24, 29) and (b) its social media literacy training for a wider circle of civil society (Interview 21, 28). As for Thailand, besides Twitter’s and Facebook’s removal of IO networks in 2021, the social media giants have not actively addressed other criticisms on its shortcomings in transparency, remedy mechanisms, content moderation, or defence of dissident voice. The companies’ engagement with civil society has been mainly in the form of low-cost fixes, such as social media literacy training by Google, Meta, and TikTok (Interview 33; Asia Centre, 2023c) or Meta’s policy consultation sessions with selected civil society organisations (Interview 5, 7). Moreover, since 2022, Meta’s policy consultation or direct channel for Thai Trusted Partners to escalate violations has stopped and there has not been similar engagement by X or YouTube either (Interview 5, 7, 33, 34). This is particularly concerning as anti-dissident abusive accounts continue to proliferate across the Big Tech platforms (Interview 7, 33).

Our social media analysis further corroborates the significance of international allies’ advocacy amplification. Myanmar advocates’ more visible international campaigns are correlated with Meta’s more aggressive effort in moderating abusive content and active banning of authoritarian accounts. Hence, the number of Myanmar authoritarian Facebook accounts and keywords we were able to collect in early 2024 is lower than that from Thailand or Cambodia. This is despite the fact that (a) Myanmar Facebook used to be the exemplar case of harbouring politically abusive content in the region and (b) we have more team members with expertise in Myanmar’s platform-mediated politics and conducted more interviews with a wider range of respondents from Myanmar. As a result, the number of captured Myanmar posts were also significantly lower than Thai or Cambodian posts. Below is the final number of keywords, accounts, and number of captured public posts across the three cases ( Table 1).

**Table 1.** Number of Facebook accounts, keywords, and posts we monitored and captured.

Country	Page	Groups	Keywords	Total number of posts
Myanmar	7	6	66	17,072
Thailand	36	2	149	53,964
Cambodia	30	1	89	161,217

**Figure 4.**
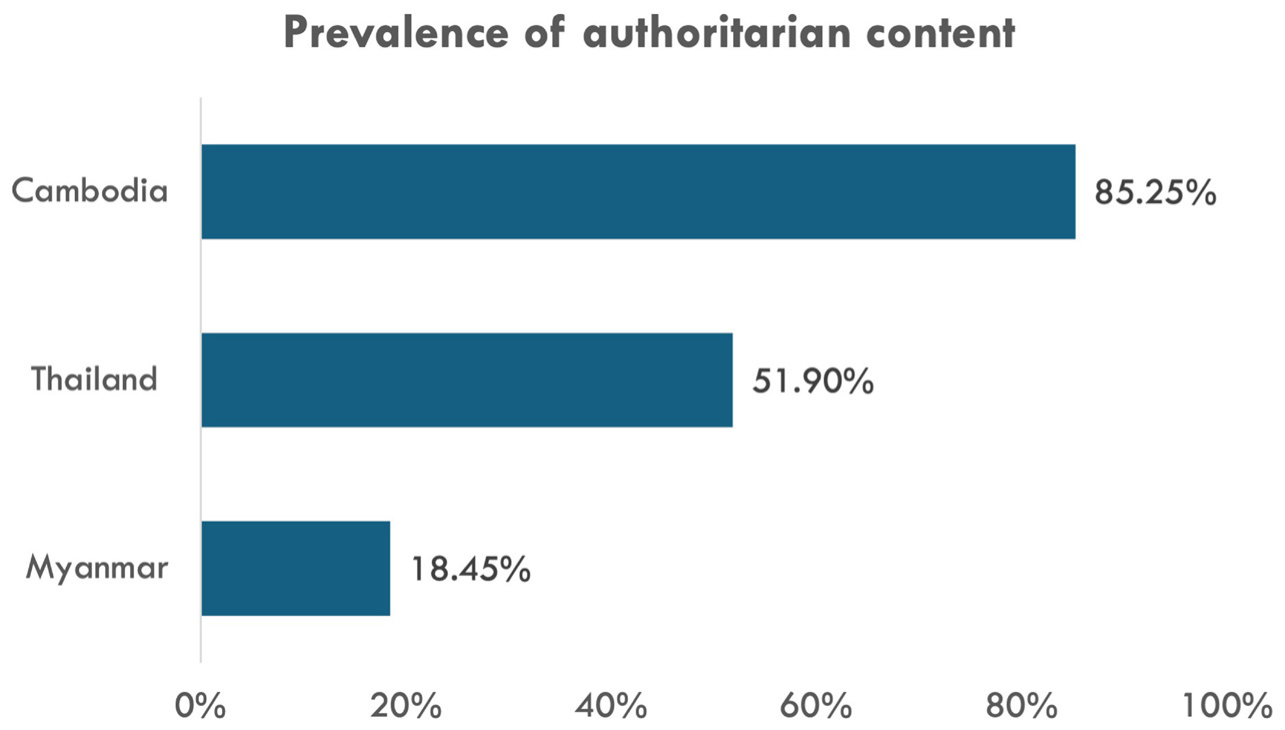
Prevalence of authoritarian content in our random samples.

As our expert interviews demonstrate, Meta has invested the least in content moderation in Cambodia. Consequently, not only did authoritarian accounts and keywords from Cambodia generate the highest number of posts ( Table 1) but our team’s iterative manual coding process also found more authoritarian content in the Cambodian sample ( Figure 4). This exhibits how much more repressed Cambodia’s social media environment is compared to Thailand and Myanmar.

The overwhelming majority of the authoritarian content belongs to the pro-regime propaganda category ( Table 2).

**Table 2.** Types of authoritarian content in our random samples.

Country	Pro-regime propaganda	Anti-dissident disinformation	Anti-dissident violence promotion	Anti-dissident doxing
Myanmar	98.92%	44.17%	22.22%	1.36%
Thailand	87.28%	30.06%	1.35%	1.06%
Cambodia	99.94%	1.06%	0.12%	0.06%

**Figure 5.**
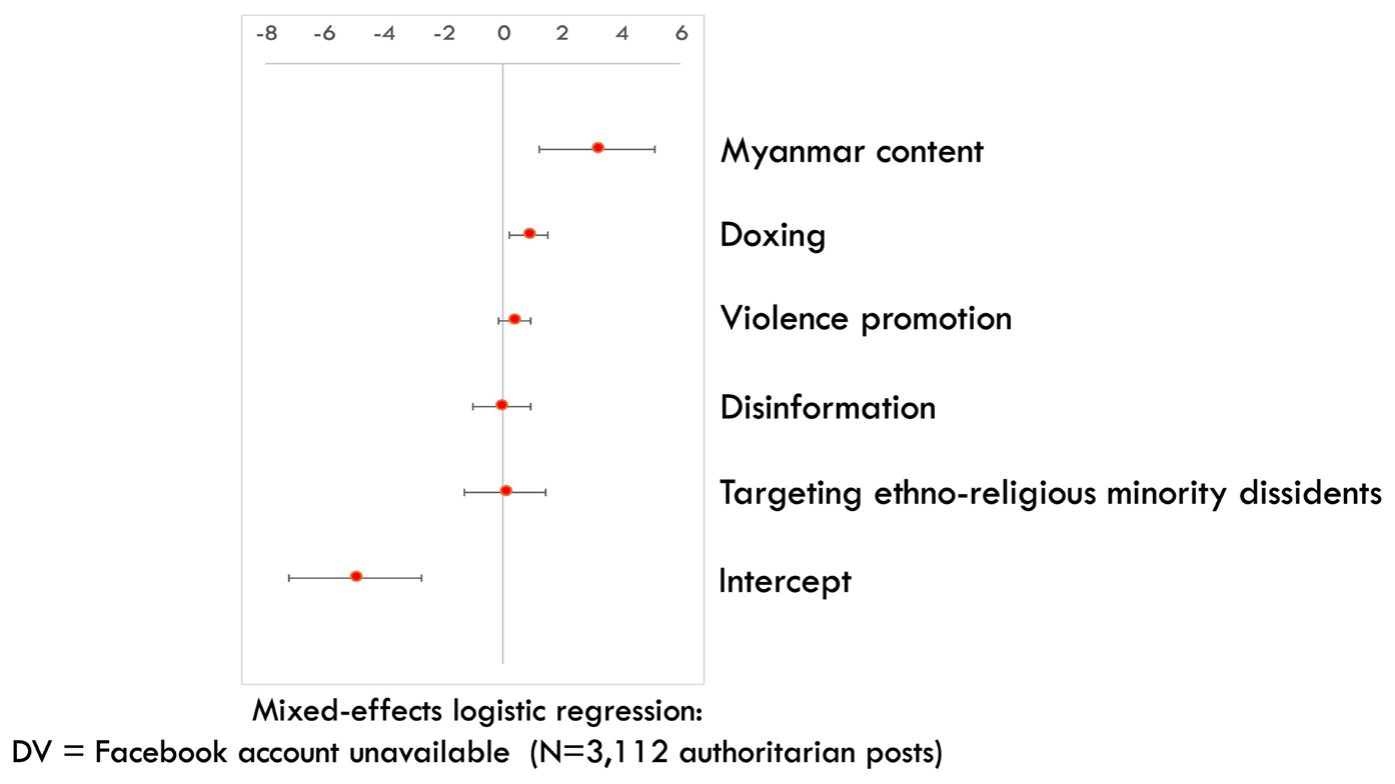
Mixed-effects logistic regression on the removal rate of authoritarian Facebook accounts across our samples (CI=95%).

Last but not least, as Myanmar is the only among the three cases where Meta has established a policy of banning regime-affiliated accounts, we expect that Myanmar accounts that have posted authoritarian content are more likely to be removed by Meta afterwards, compared to non-Myanmar accounts. To examine this proposition, we run a mixed-effects logistic regression, an approach that allows us to model clustered data, on all authoritarian posts in our coded dataset (N=3112). We cluster the posts by country cases (i.e. Myanmar, Thai, or Cambodian) and take into account case-specific sampling weights (i.e. the number of case-specific posts in the larger dataset that each post in the coded dataset represents). The outcome variable is whether the account that authored an authoritarian post during March – April 2024 is still available or unavailable as of August 2024. The deletion of pro-regime accounts is highly likely due to Meta’s removal instead of self-censorship ( Ryan & Tran, 2024). The main explanatory variable is whether the posted authoritarian content is Myanmar content or not. We further control for other potentially relevant causal factors for account removal, i.e. types of posted content that are likely to violate Facebook’s Community Standards, including: doxing, violence promotion, political disinformation, and targeting of ethno-religious minorities. The regression was conducted using Stata version 14.2 ( StataCorp, 2015). We find that accounts that have posted Myanmar content are more likely to be removed ( Figure 5) ( Tran *et al.*, 2024). This provides another probable indication of Meta’s more active approach in curbing digital authoritarian operation in Myanmar (compared to Thailand or Cambodia), a Global South case where platform advocacy efforts enjoy more attention and amplification by prominent international allies.

### Local embeddedness and representation of marginalised voices

Turning our analysis to the domestic dynamics of platform advocacy, we find that across the cases, challenges remain for the advocacy efforts to engage traumatised, marginalised targets of platform-enabled repression. A veteran human rights advocate in Cambodia admits her advocacy mainly represents the experiences of those who are either within her trusted networks or proactively reaching out to her organisation: “I think there are still a significant number of children, women, men that are being harassed at all degrees that are not going to our office. [...] The ones that we were able to tell their stories are quite courageous” (Interview 6).

Indeed, due to their long-term experience with social and political oppression, the more marginalised the dissidents are, the less confidence they have in the likelihood of Global North Big Tech platforms taking seriously their experiences with online harm. Hence, they are more reluctant to engage in transnational advocacy campaigns that will cost them physical effort, psychological toll, or maybe even retaliation by the autocrats. A Thai digital rights expert shared the difficulty she faced when trying to document cases of digital harassment: “Those who are very traumatised [by online abuse], they don’t want to talk about it at all” (Interview 34). Similarly, a Myanmar women’s rights advocate elaborated on a popular perception among female targets of repression, about platform advocacy being futile and risky efforts:

We don’t know which hate speech Facebook recognises. These types of [really sexist] content spread around the Myanmar community and didn’t get removed from Facebook. [...] Some women confirmed that many people reached into their Messenger and threatened them in different forms. But they didn’t tell how they were threatened or the exact words – for their own security. (Interview 18)

Moreover, in the Myanmar case, there exists another layer of physical challenge for its human rights advocates, most of whom have fled the country since 2021, to actively cultivate rapport with marginalised dissidents: “It could be difficult to build trust among our network members, or between the victims and our group representatives online while documenting human rights violation cases. Building trust with physical interactions on the ground is much easier” (Interview 17). As a result, even for marginalised dissidents who want to engage in platform advocacy, they lack personal connections with digital rights advocates to enable their participation (Interview 1, 11, 18, 20).

**Figure 6.**
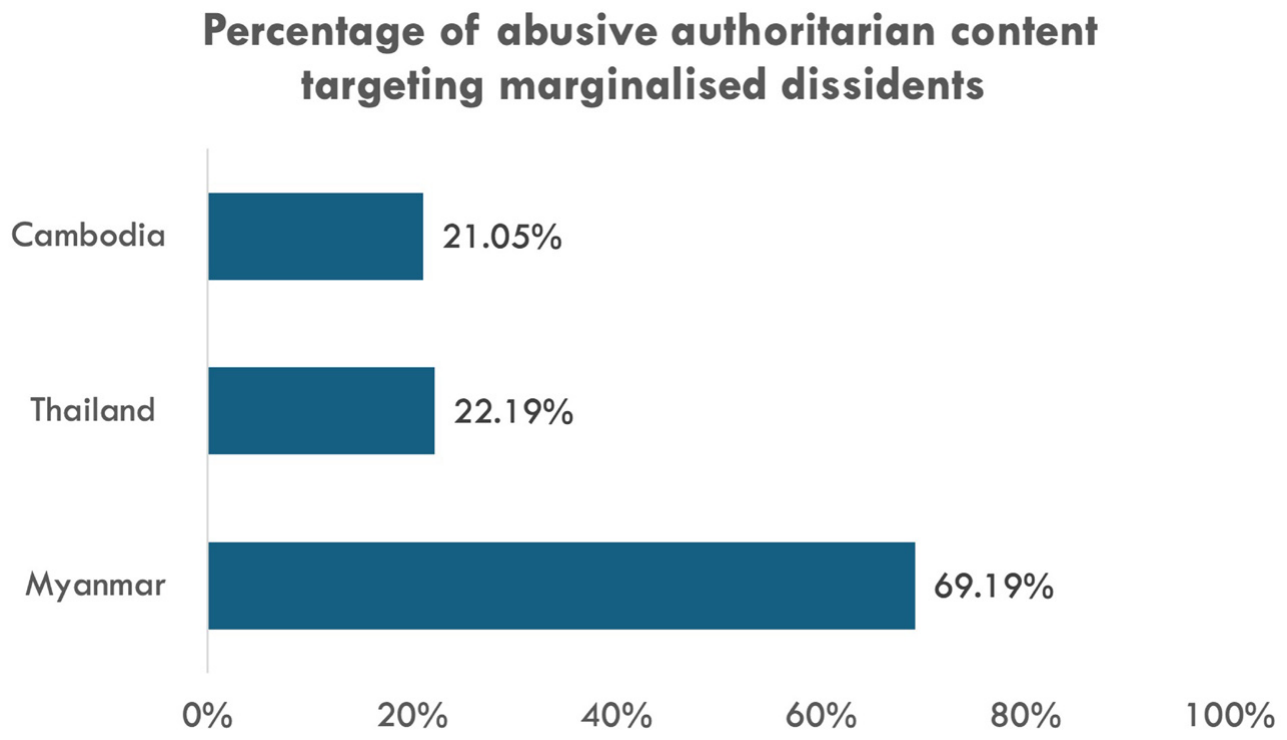
Percentage of content targeting marginalised dissidents among abusive authoritarian content.

Our social media analysis corroborates the qualitative findings. Across the three cases, there is no significant evidence of accounts targeting marginalised dissidents being more or less likely to be removed compared to other types of authoritarian accounts ( Figure 4). This demonstrates that when Facebook took action against accounts targeting marginalised dissidents, it is likely because of the abusiveness of the content instead of the targets’ identities. In the case of Myanmar, this identity-blind approach to content moderation might have facilitated a proliferation of authoritarian content against marginalised dissidents in absolute terms. In particular, among abusive authoritarian content (disinformation, violence promotion, or doxing), content targeting marginalised dissidents is the overwhelming majority in the Myanmar case (almost 70%) compared to its neighbours (around 20%) ( Figure 6). Around 93% of such Myanmar content targets ethno-religious minorities. This suggests anti-dissident forces in Myanmar still find Facebook to be a conducive platform to promote their existing divisive agenda despite the platform’s increased effort in content moderation in general. Indeed, the finding underscores marginalised dissidents’ limited ability in demanding Big Tech’s dedicated measures to ensure safer, more inclusive online spaces, particularly in the case of Myanmar.

Overall, based on original data from Myanmar, Thailand, and Cambodia, we find that pro-democracy platform advocacy depends largely on Western funding and global amplification in order to influence social media giants’ content policy. First, our mixed methods analysis of expert interviews, platform advocacy campaigns, and Meta's content moderation actions demonstrate that advocacy efforts that focus on resonant issues with Western priorities in platform governance have been much more likely to retain Western funding and attract attention from internationally prominent advocates for human rights. Second, our interviews and social media analysis further confirm the important role of amplification by the Southeast Asian advocates’ prominent allies abroad in pressuring tech giants into limiting authoritarian operations on their sites. Third, complementary insights from the qualitative interviews and social media data indicate a correlation between marginalised dissidents’ lack of in-person trust-building interactions with advocacy actors and their sustained exposure to online abuse compared to other dissidents. In the next section, we discuss our findings’ implications for the scholarship on platform governance and transnational advocacy and then conclude.

## Discussion and conclusion

As the haphazard practice by foreign tech giants increasingly shapes the landscape of civic space across democratic and authoritarian societies alike, human rights advocates have intensified their efforts to make demands on platform accountability. The advocacy might include calling on platforms to implement supportive mechanisms for vulnerable users, amplify ethical journalism, provide more transparency on moderation processes and responses, and invest more resources in smaller markets or politically unstable societies. Our theoretical framework already suggests how platform governance in the Global South might diverge from the Global North context, especially with regard to platform-enabled repression of dissident voices. The empirical findings corroborate our expectations by underscoring (a) the suppression of Global South demands that contradict Western priorities in platform governance, (b) the importance of campaign promotion by influential international allies not only for advocacy effectiveness but also for physical security of Global South human rights advocates, and (c) the vulnerability of dissidents under multiple layers of marginalisation. These findings enrich the platform governance literature by illuminating how human rights advocates in Southern autocracies have to overcome unique challenges compared to their Northern counterparts when attempting to influence Big Tech’s practice. 

First, Western governments still play the main role in funding and sustaining Global South advocacy efforts. As digital rights issues are relatively new, they have not been well incorporated in these governments’ development aid agenda (i.e. UN Sustainable Development Goals). As a result, Global South digital rights advocates face additional challenges in calling for Western funding. Such challenges are exacerbated when the advocacy issues do not resonate with demands promoted by Western democracies (see Ong *et al.*, 2024). Specific cases of platform advocacy from Myanmar and Thailand demonstrate that Global South demands that asked platforms not to promote election, to reconsider foreign interference policy, not to censor graphic violence, or to challenge government request were less likely to attract funding and support by foreign actors. This resulted in Meta’s lack of effort in addressing these demands. Our finding problematises a conventional assumption on the tendency of Southern human rights demands to find sympathetic ears among Western promoters of liberal politics ( Keck & Sikkink, 1998). On the other hand, as our quantitative analysis finds, demands that resonate with Western actors, such as improving moderation of political disinformation, received much more Western support and, hence, active response by Meta. Hence, our research enriches the latest debates in the transnational advocacy scholarship by uncovering a likelihood for conflicting pro-democracy agendas between Global North and Global South civil society in an age of Big Tech-mediated politics.

Second, as pro-democracy platform advocacy efforts focus on Big Tech’s practice in the Global South, Western governments might have lower willingness or capacity to regulate and threaten Big Tech with punishment. Instead, other non-state actors such as internationally prominent media and human rights advocates who are more sympathetic to the cause of Global South dissidents play a more important role in incentivising Big Tech to accommodate the dissidents’ demands. Our qualitative analysis of pro-democracy platform advocacy efforts in Southeast Asia finds that only after these international allies promoted local documentation of Big Tech-enabled repression, did these tech giants take costly actions to address the corresponding issues. Examples of their costly responses include TikTok’s recruitment of Burmese-speaking ^3^ moderators and start of engagement with local human rights advocates, Telegram’s more active removal of anti-dissident abusive Myanmar content and accounts, and Meta’s noncompliance with Thai authorities’ takedown request against a dissident account. Our quantitative social media analysis further demonstrates that Myanmar advocates’ more visible international campaigns have resulted in Meta’s more aggressive effort in moderating authoritarian actors in early 2024 compared to Thailand and Cambodia. This finding generates a fresh perspective on transnational advocacy by demonstrating that while Big Tech might be less exposed to Western sanctions than Global South autocrats, they appear to be more susceptible to international naming and shaming. These profit-driven entities might have more to lose as their global reputational damage could trigger negative market reactions ( De Winter, 2001, 108; Pye, 2023, 336). Hence, this key aspect might distinguish platform advocacy from the conventional state-centric advocacy framework. 

Third, due to the lack of Big Tech’s physical presence compared to other Global North multinational corporations operating in Southern markets, human rights advocates with experience in transnational advocacy play a more substantial role in shaping platform advocacy efforts. Indeed, advocacy around other multinationals’ labour abuse or environmental destruction in the Global South often features public protests by aggrieved workers or local residents, whose representatives are deeply involved in negotiations, advocacy, or litigation toward the Global North corporations. On the other hand, platform advocacy around Big Tech-enabled repression usually lacks visible participation by the actual repression victims. Hence, there is a higher risk of the advocacy’s lack of representation of marginalised voices. Our interviews with advocacy actors and dissidents from Southeast Asia find that fear of autocrats’ retaliation has particularly discouraged marginalised victims to engage in transnational advocacy campaigns. The social media analysis further suggests that challenges remain in advocacy against platform-enabled repression of the most marginalised. This finding nuances our understanding on the role of political regime on transnational advocacy ( Zajak, 2017, 139): while repression might not inhibit advocacy campaigns (as Myanmar, the most violently repressive case, witnesses the most active platform advocacy), it might hinder the inclusiveness of advocacy demands.

Overall, by conducting a novel mixed methods study on the dynamics of pro-democracy platform advocacy across three comparative Southeast Asian cases, we find that such advocacy is more likely to curtail Big Tech-mediated repression when advocacy actors focus on demands that resonate with Western democracies’ agenda in platform governance, engage prominent international allies to amplify their demands, and represent the experiences of marginalised dissidents. The findings make important contributions to both the platform governance and transnational advocacy scholarship by underscoring the unique dynamics of Big Tech governance under authoritarianism in the Global South. Methodologically, by strictly limiting the scope of social media processing to publicly available content with carefully selected accounts and keywords, this study showcases a promising big-data design that minimises privacy risks to vulnerable social media users.

To advance our knowledge frontier on Big Tech governance in Global South authoritarian contexts, we will benefit from future research that scrutinises the dynamics of South-South collaboration in transnational platform advocacy efforts in Southeast Asia and beyond. Different from Hall’s (2022) finding on Global North transnational advocacy where digitalisation has enabled more cross-case collaboration, our interviews indicate that high-level collaboration among Global South human rights advocates is rather limited. Most collaborations stay at the level of signing one another’s petitions ( Global Digital Justice Forum, 2025). It is essential to understand factors that moderate South-South collaboration. Another important venue to explore is how researchers can benefit from new regulatory mechanisms, such as the EU’s Digital Services Act, to access traditionally undisclosed data on Big Tech’ content moderation practice in order to corroborate existing findings based on social media monitoring and platforms’ own transparency reports.

## Ethics and consent

The research obtained ethical approval by the Vrije Universiteit Brussel’s Human Sciences Ethics Committee before conducting interviews (Reference number: ECHW_459.02-WP2, approval date: 13 November 2023) and collecting social media content (Reference number: ECHW_459-WP2.02, approval date: 18 December 2023).

Each interviewee provided verbal consent before their interview started. The interviewees might be deterred from participating in the research if they need to sign a form with full details about the research. Their signature is a type of personal identification that directly ties them to the research described in the form. Hence, to ensure privacy and confidentiality and allow for smooth research implementation, we only obtained their verbal consent. The verbal consent procedure is approved by the ethics committee (Reference number: ECHW_459.02-WP2, approval date: 13 November 2023).

Consent for collecting social media content is waived by the ethics committee (Reference number: ECHW_459-WP2.02, approval date: 18 December 2023). As acquiring consent is practically unfeasible due to the use of large quantities of publicly available data, the processing of data is based on the public interest regarding academic scientific research serving the advancement of general knowledge to the advantage of society, as recognized in the Flemish Codex Hoger Onderwijs. Hence, the research did not draw up an informed consent form but published a Privacy Policy on the project’s website. Moreover, participants’ risk is mitigated through anonymisation of published data so that no personal details are part of the finished research.

## Data Availability

Personal data are processed according to the principles stipulated by the new European General Data Protection Regulation (GDPR) that was enforced on 25 May 2018. To ensure privacy and confidentiality of vulnerable participants, only the corresponding researcher, Mai Van Tran, has access to the personal data collected (including pseudonymised interview transcripts, interviewees’ identifying information, social media posts, and the posts’ identifying information such as URLs, authors’ names, or channels’ names). Personal data from the informed consent forms are also redacted, including information on: the researcher’s phone number, secure storage of interview data, and translation support for non-English speakers. Authorised persons will need to request access from the corresponding researcher who will identify and grant individual access to people covered in a Data Processing Agreement. Zenodo: *Pro-democracy platform advocacy: A dataset of project-related content*. https://doi.org/10.5281/zenodo.14471006 ( Tran *et al.*, 2024). The project contains the following underlying data: ProPA_coded sample_anon.csv (An anonymised dataset of metadata and coding on public Facebook content). ProPA_keywords.pdf (List of keywords used to search for public authoritarian content on Facebook) The data is deidentified prior to uploading according to the Safe Harbour method. Data are available under the terms of the Creative Commons Attribution 4.0 International license (CC-BY 4.0). Zenodo: *Pro-democracy platform advocacy: A dataset of project-related content*. https://doi.org/10.5281/zenodo.14471006 ( Tran *et al.*, 2024). The project contains the following extended data: ProPA_codebook.pdf (Codebook for the dataset: ProPA_coded sample_anon.csv) ProPA_informed consent_English_redacted.pdf (Informed consent document in English, provided to English-speaking expert interviewees before their interviews) ProPA_informed consent_Khmer_redacted.pdf (Informed consent document in Khmer, provided to Khmer-speaking expert interviewees before their interviews) ProPA_informed consent_Burmese_redacted.pdf (Informed consent document in Burmese, provided to Burmese-speaking expert interviewees before their interviews) ProPA_interview guide.pdf (Interview guide for the study’s expert interviews) ProPA_privacy policy.pdf (Privacy Policy on the study’s handling of personal data from social media) ProPA_melogit.do (Do file with Stata code for running the study’s mixed-effects logistic regression) ProPA_melogit_output.pdf (Output data of ProPA_melogit.do) Data are available under the terms of the Creative Commons Attribution 4.0 International license (CC-BY 4.0).

## Software availability statement

The mixed-effects logistic regression in this article was performed using Stata version 14.2, a proprietary statistical software ( https://www.stata.com/). For those without access to Stata, equivalent analyses can be performed using open-source alternatives such as R ( https://cran.r-project.org/).
