# Supplementary material for: Pro-democracy platform advocacy: Resisting Big Tech-mediated authoritarianism in Southeast Asia
Source: Open Res Eur. 2025 May 8;5:10. Originally published 2025 Jan 20. [Version 2] doi: 10.12688/openreseurope.18820.2 (PMC12084801; doi:10.12688/openreseurope.18820.2)
Supplement: Peer review history

# Reviewer report: Reviewer response for version 2

**DOI:** 10.21956/openreseurope.22013.r54004

**Published:** 2025-05-16

**Authors:** Tawseef Majeed

**Affiliations:** 1 Jamia Millia Islamia, New Delhi, India

The author/s has addressed most comments, and the document manuscript has improved. Given the context of the article's existing literature, the trajectory of the Global South's pro-democracy activism could be explored further. Also, there could be more clarity on how Big Tech's digital dominance is significant towards pro-democracy activism. It would establish the findings more concretely. Most of the findings are robust in the context of contemporary technology and the capitalism-assisted shift in media production and consumption. This could be explored further within the prospects of digital convergence and divergence. 

 Anyways, I am glad that the article is well designed to create a robust scholarly discourse on pro-democracy advocacy in an age of Big Tech oligopoly. All the best!

If applicable, is the statistical analysis and its interpretation appropriate?

Yes

Is the study design appropriate and is the work technically sound?

Yes

Is the work clearly and accurately presented and does it engage with the current literature?

Partly

Are the conclusions drawn adequately supported by the results?

Partly

Are sufficient details of methods and analysis provided to allow replication by others?

Partly

Are all the source data and materials underlying the results available?

Partly

Reviewer Expertise:

Journalism, political communication, solutions-oriented communication, communication design

I confirm that I have read this submission and believe that I have an appropriate level of expertise to confirm that it is of an acceptable scientific standard.

# Reviewer report: Reviewer response for version 1

**DOI:** 10.21956/openreseurope.20366.r50746

**Published:** 2025-03-20

**Authors:** Verica Rupar

**Affiliations:** 1 Auckland University of Technology, Auckland, New Zealand; 2 Media Diversity Institute, London, UK

**Peer Review: Pro-democracy Platform Advocacy: Resisting Big Tech-mediated Authoritarianism in Southeast Asia**

 This research makes a significant and original contribution to the study of human rights advocacy, digital repression, and platform governance in Southeast Asia. By investigating how civil society actors engage in platform advocacy, the study fills a crucial gap in existing research. By focusing on how advocacy efforts can shape platform governance and potentially curb digital repression, it provides a compelling perspective on the power and limitations of civil society engagement with major technology companies. This work is particularly valuable given the increasing role of digital platforms in authoritarian governance.

 The study employs a novel mixed-methods approach, combining qualitative and quantitative analyses. The dataset is impressively rich, drawing on 38 semi-structured expert interviews, 6,000 Facebook posts, and Meta’s Transparency Reports. This comprehensive methodological design ensures a robust examination while also mitigating privacy risks for vulnerable social media users. The use of big-data analytics, particularly the careful curation and coding of social media content, represents a promising advancement in research on digital repression and civil society advocacy.

 The study presents compelling evidence that platform advocacy is most effective under three key conditions: when advocates align their demands with the governance priorities of Western democracies; when they collaborate with influential international allies to amplify their message; and when they center the experiences of marginalized dissidents, such as female, LGBTQ+, and ethno-religious minority activists. These findings provide critical insights into the mechanisms through which advocacy can challenge digital repression and influence platform policies.

 By examining platform advocacy in Myanmar, Thailand, and Cambodia, three countries where autocrats frequently exploit social media for political control, the study effectively highlights both the shared and divergent challenges faced by pro-democracy activists in these contexts, making a strong case for the need for regionally and globally coordinated advocacy efforts.

 The findings have direct implications for policymakers, technology companies, international media, and human rights organizations. The study underscores how global tech firms' inconsistent and often haphazard governance practices shape civic spaces in authoritarian contexts. It also highlights the need for greater accountability in content moderation, investment in protective mechanisms for vulnerable users, and transparency in platform decision-making. The research further demonstrates the vulnerabilities of Global South advocacy efforts, particularly the suppression of demands that conflict with Western platform governance priorities and the critical role of international allies in securing both advocacy impact and activists' physical safety.

 The paper makes an outstanding contribution to the scholarship on digital repression, civil society advocacy, and platform governance. Its rigorous methodology, rich empirical data, and comparative insights provide a crucial foundation for future research and policy discussions on pro-democracy activism in the digital age. The findings are not only academically valuable but also have practical implications for human rights defenders, technology companies, and global policymakers.

 Overall, this is a well-researched, timely, and impactful study that advances our understanding of the role of digital platforms in both enabling and constraining democratic participation in Southeast Asia.

If applicable, is the statistical analysis and its interpretation appropriate?

Yes

Is the study design appropriate and is the work technically sound?

Yes

Is the work clearly and accurately presented and does it engage with the current literature?

Yes

Are the conclusions drawn adequately supported by the results?

Yes

Are sufficient details of methods and analysis provided to allow replication by others?

Yes

Are all the source data and materials underlying the results available?

Yes

Reviewer Expertise:

Journalism studies, media and diversity, media and politics

I confirm that I have read this submission and believe that I have an appropriate level of expertise to confirm that it is of an acceptable scientific standard.

# Reviewer report: Reviewer response for version 1

**DOI:** 10.21956/openreseurope.20366.r50742

**Published:** 2025-03-18

**Authors:** Tawseef Majeed

**Affiliations:** 1 Jamia Millia Islamia, New Delhi, India

I appreciate the research study undertaken by the authors; it is contemporaneously relevant and has the potential to contribute to a precise understanding of Big Tech-mediated authoritarianism in Southeast Asia. However, I offer a few suggestions to strengthen the study's primary arguments. 

 The literature review needs more engagement with contemporary pro-democracy advocacy and authoritarianism; the conceptualization is a little weaker. This can be included in the theorization section of the article. The authors could navigate through the more enormous democracies in South-East Asia. Also, the authors can contextualize the concepts with a broader understanding of democracy and authoritarianism in the Asian context (India, Pakistan, China, and more). 

 The methodology section looks reasonably sound. However, I suggest tabulating the main findings and testimonies of the interview data. It will look more organized and enhance the data presentation of the article. 

 Furthermore, the discussion and conclusion section needs to engage more robustly with the main findings. It has the potential to make it more academic. 

 The article is interesting. The authors' efforts are visible throughout the manuscript, and I enjoy reading it. I wish them all the best.

https://www.journalofdemocracy.org/articles/the-authoritarian-roots-of-indias-democracy/

https://chomsky.info/20180725/

If applicable, is the statistical analysis and its interpretation appropriate?

Yes

Is the study design appropriate and is the work technically sound?

Yes

Is the work clearly and accurately presented and does it engage with the current literature?

Partly

Are the conclusions drawn adequately supported by the results?

Partly

Are sufficient details of methods and analysis provided to allow replication by others?

Partly

Are all the source data and materials underlying the results available?

Partly

Reviewer Expertise:

Journalism, political communication, solutions-oriented communication, communication design

I confirm that I have read this submission and believe that I have an appropriate level of expertise to confirm that it is of an acceptable scientific standard, however I have significant reservations, as outlined above.

## References

[1] : Embedded authoritarianism: the politics of poor press freedom in Indian Kashmir. *Media, Culture & Society* .2022;44(3) : 10.1177/01634437211069973 613-624 10.1177/01634437211069973

[2] : The structural origins of authoritarianism in Pakistan. *Commonwealth & Comparative Politics* .2013;51(1) : 10.1080/14662043.2013.750064 122-142 10.1080/14662043.2013.750064
